# Diluted Aqueous Dispersed Systems of 4-Aminopyridine: The Relationship of Self-Organization, Physicochemical Properties, and Influence on the Electrical Characteristics of Neurons

**DOI:** 10.3389/fchem.2021.623860

**Published:** 2021-03-16

**Authors:** Irina Ryzhkina, Lyaisan Murtazina, Khalil Gainutdinov, Alexander Konovalov

**Affiliations:** ^1^ Arbuzov Institute of Organic and Physical Chemistry, FRC Kazan Scientific Center, Russian Academy of Sciences, Kazan, Russia; ^2^ Zavoisky Physical-Technical Institute, FRC Kazan Scientific Center, Russian Academy of Sciences, Kazan, Russia

**Keywords:** 4-aminopyridine, diluted dispersed systems, self-organization, *Helix lucorum* snail, pH of systems, electrical conductivity, fluorescence, membrane potential

## Abstract

A variety of physicochemical methods were used to examine the self-organization, physicochemical, UV absorption, and fluorescent properties of diluted aqueous solutions (calculated concentrations from 1·10^−20^ to 1·10^−2^ M) of the membrane voltage-dependent potassium channels blocker 4-aminopyridine (4-AP). Using the dynamic light scattering method, it was shown that 4-AP solutions at concentrations in the range of 1·10^−20^–1·10^−6^ M are dispersed systems in which domains and nanoassociates of hundreds of nm in size are formed upon dilution. An interrelation between the non-monotonic concentration dependencies of the size of the dispersed phase, the fluorescence intensity (*λ*_ex_ 225 nm, *λ*_em_ 340 nm), specific electrical conductivity, and pH has been established. This allows us to predict the bioeffects of the 4-AP systems at low concentrations. The impact of these diluted aqueous systems on the electrical characteristics of identified neurons of *Helix lucorum* snails was studied. Incubation of neurons in the 4-AP systems for which the formation of domains and nanoassociates had been established lead to a nonmonotonic decrease of the resting potential by 7–13%. An analysis of the obtained results and published data allows for a conclusion that a consistent change in the nature and parameters of the dispersed phase, as well as the pH of the medium, apparently determines the nonmonotonic nature of the effect of the 4-AP systems in a 1·10^−20^–1·10^−6^ M concentration range on the resting membrane potential of neurons. It was found that the pre-incubation of neurons in the 4-AP system with a concentration of 1·10^−12^ M led to a 17.0% synergistic decrease in the membrane potential after a subsequent treatment with 1·10^−2^ M 4-AP solution. This finding demonstrates a significant modifying effect of self-organized dispersed systems of 4-AP in low concentrations on the neurons’ sensitivity to 4-AP.

## Introduction

Studies of the phenomena of nonmonotonic concentration dependences of the bioeffects of diluted aqueous solutions of biologically active substances (BAS) are of current interest (Mark and Edward, 2009; Shimanovsky et al., 2010; Tarasova and Makarova, 2016). The demand in such studies is due to the problem of significant side effects arising from the use of drugs by the population in high doses. Reduction in the therapeutic dose is one of the ways to improve drug tolerability and reduce their negative effects on the organism. It is thought that lower doses can ensure drug selectivity and make it possible to avoid adverse side effects with a comparable therapeutic efficacy (Mark and Edward, 2009; Shimanovsky et al., 2010).

However, it has been established that the effect of many drugs at low concentrations can significantly differ from their effects at therapeutic doses, both in the direction of the effect and in the mechanism of action. Pharmacological profiles of solutions of therapeutic substances are usually complex within the range of low concentrations and characterized by a number of peculiarities: nonmonotonic dose-effect relationship, existence of the “silence zones” where biosystems are practically insensitive to the substance, and change in the direction of the bioeffect, etc. (Mark and Edward, 2009; Shimanovsky et al., 2010). The experimentally established synergism of action of the solutions prepared with high and low concentrations of the same substance (i.e., a more pronounced effect of a combination of such solutions than the simple sum of the effects of each of them) can be used as the basis for increasing of efficiency, reduction of toxicity, as well as the possibility of expansion of the action spectrum of the drugs (Marotta et al., 2003; Pal'mina et al., 2003).

Obviously, fundamental knowledge, elucidation of the mechanism of the effect of dilute aqueous solutions with low concentrations of solutes on living systems, and the substantiation and prediction of their bioeffects is required for the scientific explanation of the observed phenomena. Physicochemical research of diluted systems is well covered (Tournier et al., 2019), but the vast majority of works do not use physicochemical studies to explain the cause of bioeffects in dilute solutions, do not study the relationship between self-organization and bioeffects, and do not attempt to predict bioeffects based on physicochemical experimental data.

Currently, a new physicochemical approach is being developed that explains and predicts the presence of bioeffects in water systems with low calculated concentrations^1^ of substances and nonmonotonically dependent bioeffects on concentration (Konovalov and Ryzhkina, 2014; Ryzhkina et al., 2018a; Ryzhkina et al., 2018b; Ryzhkina et al., 2019; Ryzhkina et al., 2020).

It has been found that the diluted aqueous solutions of various BAS are open self-organized dispersed systems capable of formation and rearrangement of the dispersed phase of the “supramolecular domain-nanoassociate” and “nanoassociate-nanoassociate” types. Parameters of such dispersed phases alter nonmonotonically with the dilution. This is reflected by a coherent change in physicochemical (electrical conductivity, pH, surface tension, etc.) and biological properties of systems that are most pronounced at common critical concentrations (Konovalov and Ryzhkina, 2014; Ryzhkina et al., 2018a; Ryzhkina et al., 2018b; Ryzhkina et al., 2019; Ryzhkina et al., 2020).

It was shown that one of the main differences between supramolecular domains (Sedlák, 2006; Sedlák and Rak, 2013) and nanoassociates is that domains are usually formed at sufficiently high concentrations (from 1 M to 10^−5^ M) both in the presence and absence of the background low-frequency electromagnetic fields (EMF). Nanoassociates are formed as the solution is being diluted only in the presence of EMF, and characterized by dimensions of hundreds of nm, a negatively charged interface, and high susceptibility to such factors as the chemical and spatial structure of solute molecules, the properties of EMF, etc. (Konovalov and Ryzhkina, 2014; Ryzhkina et al., 2018a; Ryzhkina et al., 2018b; Ryzhkina et al., 2019; Ryzhkina et al., 2020).

As dilution proceeds, a coherent change in the extreme values of the parameters of nanoassociates, physicochemical properties, and bioeffects of the systems are observed at practically the same critical concentrations. This made it possible to propose a hypothesis and experimentally prove that the nonmonotonic nature of the bioeffect, a change in its direction, the presence of “zones of silence” are primarily due to the transformation of the dispersed phase in the course of the systems’ dilution, rather than the decrease of the solute’s concentration (Konovalov and Ryzhkina, 2014; Ryzhkina et al., 2018a; Ryzhkina et al., 2018b; Ryzhkina et al., 2019; Ryzhkina et al., 2020). Moreover, it follows that it is possible to predict the emergence of similar concentration dependences of bioeffects using nonmonotonous concentration dependences of nanoassociates' parameters and physicochemical properties of systems in the interval of low calculated concentrations (Konovalov and Ryzhkina, 2014; Ryzhkina et al., 2018a; Ryzhkina et al., 2018b; Ryzhkina et al., 2019; Ryzhkina et al., 2020).

Recently, and for the first time, it was established that the rearrangement of nanoassociates at certain low critical concentrations is accompanied not only by a significant change in physicochemical properties and bioeffects but also in the UV absorption in the 200–300 nm range (Ryzhkina et al., 2018a; Ryzhkina et al., 2018b). Works (Lobyshev et al., 1999; Chai et al., 2008; Elia et al., 2014; Elia et al., 2018; Patsaeva et al., 2018) have demonstrated the presence of absorption within the range of 200–350 nm in the UV spectra of natural water, aqueous solutions of BAS, and systems containing structured water in contact with a hydrophilic surface. Excitation in this region results in fluorescence within two spectral ranges: 300–350 and 400–450 nm. This allowed some authors (Lobyshev et al., 1999; Chai et al., 2008; Elia et al., 2014; Elia et al., 2018) to assume that the observed spectral properties are characteristic of the ordered water structures forming in nano-heterogeneous water systems.

A correlation has been established between a nonmonotonic change in the fluorescence intensity of highly diluted sodium chloride solutions and mobility of unicellular ciliates (Lobyshev et al., 2005). It was later found that nanoassociates form in the aqueous system of sodium chloride around concentrations at which fluorescence peaks and maximum bioeffects are observed (Ryzhkina et al., 2012).

For a deeper understanding of the nature of the discovered phenomena and their relation to real-life tasks of pharmacology and medicine, it is necessary to continue research of self-organization and physicochemistry of the diluted solutions of commonly used or currently developed drugs. In addition, it is important to expand the range of research methods by using UV absorption and fluorescence spectroscopy, paying special attention to the establishment of the relationship between the formation of nanoassociates, the physicochemical and spectral properties of the systems, and their bioeffects.

In this work, an aqueous solution of 4-aminopyridine (4-AP), a well-known blocker of potassium channels, was chosen as the object of research (Hermann and Gorman, 1981; Mathie et al., 1998; Armstrong and Loboda, 2001; Kiss et al., 2002; Gutman et al., 2005). 4-AP is a ligand and a modulator of proton-activated cationic channels of the acid-sensing ion channel (ASIC) family widely expressed in the central nervous system of vertebrates and plays an important role in physiological and pathological processes (Tikhonov et al., 2019; Serova et al., 2020). Recent studies on the mechanisms of action of potassium channel blockers and ASIC ligands have shown that they can be of great interest not only for neurophysiology but also for meeting a pharmaceutical need for new drug development (Andreani et al., 2000; Zamoyski et al., 2016; Li et al., 2017; Hassan et al., 2018).

Currently, 4-AP is used in the treatment of patients with spinal cord injury, stroke, multiple sclerosis, and other nervous system diseases (Wu et al., 2009; Namura et al., 2010; Jensen et al., 2014; Arreola-Mora et al., 2019; Rodríguez-Rangel et al., 2020). Typical daily doses of 4-AP are in the range of 10–60 mg/day (Namura et al., 2010; Jensen et al., 2014; Arreola-Mora et al., 2019). When taken at a dose of 30 mg/day, the 4-AP plasma concentration is expected to be within the range of 2.5–80 ng/mL. Patients taking 4-AP above this dose may face side effects associated with the central nervous system stimulation as well as vasospasm (Namura et al., 2010; Arreola-Mora et al., 2019).

Taking into account the wide range of applications of 4-AP, the determination of self-organizing ability of its solutions within the range of low calculated concentrations can become the key to understanding the mechanism of the 4-AP system’s action on biological objects and the development of approaches to their more effective and safe usage.

Information about the mechanisms of action of ion channel blockers, such as 4-AP, often comes from the results of electrophysiological studies of the effect of 4-AP solutions on electrical characteristics of neurons in commonly used concentrations (0·1×10^−3^–10·10^−3^ M) (Hermann and Gorman, 1981; Mathie et al., 1998; Kiss et al., 2002; Zamoyski et al., 2016; Tikhonov et al., 2019; Serova et al., 2020). Since potassium current plays a key role in the action potential generation by neurons and its propagation along axons and dendrites, 4-AP has a critical effect on nervous system functioning. In this regard, the effect of the K^+^-channel blockers on the generation of excitation in neurons is traditionally studied on identified neurons of the *Helix lucorum* snail which is a simple model of the nervous system.

The aim of this work was to investigate the self-organization, physicochemical, UV absorption, and fluorescent properties of the 4-AP aqueous systems within the range of calculated concentrations of 1·10^−20^–1·10^−2^ M, establishing the relationship between nonmonotonic concentration dependences of the size of the dispersed phase, electrical conductivity, pH, and fluorescence intensity in these systems. Also, our goal included predicting and experimentally testing the effect of 4-AP saline solutions within a wide range of the calculated concentrations on electrical properties of identified neurons of the *Helix lucorum* snail. In addition, physicochemical methods were applied for research of the systems obtained by the first and the second consecutive centesimal dilutions of the 4-AP system with a calculated concentration of 1·10^−20^ M (hereinafter referred to as S-22 and S-24, respectively) as well as the water mixed systems (MS) in which a high-concentration 4-AP solution was one of the components, and the other – the 4-AP systems with the calculated concentrations within the range of 1·10^−20^–1·10^−6^ M, and the S-24 system. To establish the modifying effect of dilute dispersed systems, we studied the electrical characteristics of neurons placed in 4-AP solution at a concentration commonly used for electrophysiological studies (1·10^−2^ M) after their preincubation in a system with a low calculated concentration of 4-AP (1·10^−12^ M) or in the S-24 system.

## Materials and Methods

### Chemicals

4-AP (purity >98%) was purchased from Sigma–Aldrich. Solutions were made in 15 mL Wiegand (120011543) vials using freshly prepared double distilled water with specific conductivity no more than 1.5 μS/cm and free of any particles as checked by a Malvern Instruments Zetasizer Nano ZSP analyzer.

### Experimental Design

To prepare solutions with low concentrations of solutes, we usually use the method of sequential decimal or centesimal dilution of the initial solution with concentrations of the substance in the range from 1 M to 1·10^−3^ M, depending on its solubility and the purpose of the study. In the case of high dilutions (less than 1·10^−12^ M), the actual concentration of the substance is rather difficult to confirm by experimental methods. Therefore, in our previous papers (Konovalov and Ryzhkina, 2014; Ryzhkina et al., 2018a; Ryzhkina et al., 2018b; Ryzhkina et al., 2019; Ryzhkina et al., 2020), as well as in the present work, when discussing high dilutions corresponding to calculated concentrations from 1·10^−13^ M to 1·10^−20^ M, we use this very term implying the theoretically possible concentration of the substance at the corresponding dilution step.

To carry out physicochemical experiments, 1 M of 4-AP stock solution was prepared from which centesimal dilutions (in a volume of 10 ml) were made using double distilled water as a solvent. For a biophysical experiment, 1·10^−2^ M of 4-AP stock solution was prepared from which centesimal dilutions (in a volume of 10 ml) were made using a physiological solution for invertebrates as a solvent. The solutions were stirred using a minishaker (Shaker lab dancer, IKA, Germany) for 10 s and kept for 20 h (Konovalov and Ryzhkina, 2014; Ryzhkina et al., 2018a; Ryzhkina et al., 2018b; Ryzhkina et al., 2019; Ryzhkina et al., 2020). Prior to the measurements, the solutions were kept at constant temperature 25 ± 0.1°C for 1 h.

We performed two series of experiments. In the first series, we studied 4-AP aqueous solutions within a wide range of calculated concentrations 1·10^−20^–1·10^−2^ M as well as the solutions S-22 and S-24 obtained by serial centesimal dilutions of the 4-AP system with the calculated concentration of 1·10^−20^ M. In the second series, we studied mixed systems consisting of two components: a standard (1·10^−2^ M) 4-AP solution and a much more diluted one in concentrations of 1·10^−6^, 1·10^−12^, 1·10^−18^ M, or S-24 system.

### Physicochemical Methods

The self-organization of the 4-AP systems were studied using the methods of dynamic light scattering (DLS) and electrophoretic light scattering (ELS) (Zetasizer Nano ZSP analyzer, Malvern Instruments, United Kingdom). The particle size (the effective hydrodynamic diameter (*d*) of the kinetically labile particles at the maximum of the distribution curve) and polydispersity index (IP) were determined on a Zetasizer Nano ZSP analyzer (Malvern Instruments, United Kingdom) equipped with a 633 nm He-Ne laser and operating at an angle of 173°. To collect and analyze the data, the Dispersion Technology Software version 7.10 from Malvern was used. Each sample was measured in single-use polystyrene cuvettes (Sarstedt, Germany) with a pathlength of 10 mm. The measurements were made at a position of 4.65 mm from the cuvette wall with an automatic attenuator and at a controlled temperature of 25 ± 0.1°C. The solutions were freed of dust by filtering through Iso-Disc N-25–4 Nylon filters (Supelco, United States). The parameters of nanoassociates *(d*, ζ-potential) and the properties of solutions of each concentration were measured in three parallel samples with a threefold repetition of each experiment (independently prepared dilution).

Changes in the electrical conductivity (χ) of the solutions at 25 ± 0.1°C were determined using a conductometer (inoLab Cond Level 1, WTW, Germany). pH was measured by a pH-meter (inoLab pH 720, WTW, Germany) at 25 ± 0.1°C.

Statistical analysis was performed with the Student's t-test using Microsoft Excel software at a statistical significance level of 5%. The range of measurement errors of the nanoassociates’ parameters and physicochemical properties of the solutions (specific conductivity, pH) varied depending on the dilutions. At high concentrations of the system, measurement errors were within a 2–5% range. At further dilutions, the errors varied nonmonotonically from 5 to 20%. Similar to other research (Konovalov and Ryzhkina, 2014; Ryzhkina et al., 2018a; Ryzhkina et al., 2018b; Ryzhkina et al., 2019; Ryzhkina et al., 2020), all studies were carried out at the temperature of 25 ± 0.1°C.

The UV spectra of the solutions were obtained using a UV/Vis Spectrophotometer Cary 100 (Agilent, United States), with a spectral range of 190–900 nm, and a wavelength accuracy and repeatability of <±0.02 and <0.008 nm, respectively. The spectra were recorded at a scan rate of 600 nm/min and 1 nm scan step. We used QS-SUPRASIL quartz cuvettes with a 1 cm optical path.

As a reference sample for the UV absorption spectra recording, we used the same water which was used as a solvent for the preparation of test samples. The spectra were measured many times, no differences in the spectra of the samples prepared in parallel were found.

Fluorescence spectra were recorded with a Cary Eclipse fluorescence spectrophotometer (Agilent, United States). The spectra were recorded with a monochromator excitation and emission slit width of 5 nm at 25°C. We used standard (10 mm) quartz cuvettes for fluorescence (Part # 6610000900, Agilent technologies, Germany). The cuvettes were thermostated with a Peltier element. We investigated the fluorescence spectra at the excitation wavelength (*λ*_ex_) of 225 nm as well as excitation spectra at the emission wavelength (*λ*_em_) of 340 nm. Intensity value at the emission band maximum was used as the measure of fluorescence intensity. The discrepancy between the results of parallel experiments did not exceed 20%.

### Biophysical Method: Influence of the 4-AP Systems on Electrical Characteristics of Command Neurons

The effect of the 4-AP systems with a wide range of concentrations on electrical characteristics (EC) of neurons was analyzed using the premotor interneurons LPa3, RPa3, LPa2, and RPa2 of a terrestrial snail (*Helix lucorum*). For this, a ganglion nerve ring was placed in the saline solution (SS) for invertebrates of the following composition (mM): NaCl - 78, KCl - 4.5, NaHCO_3_ - 4.5, MgCl_2_ - 6.7, CaCl_2_ - 10.

We conducted two series of experiments:

1) The first series: we assessed the effect of saline solutions of 4-AP within a wide range of calculated concentrations (1·10^−20^–1·10^−2^ M) and saline solutions S-22 and S-24 on the EC of the studied neurons. For each neuron, EC was recorded twice: before the exposure (neuron in saline) and after 30 min of 4-AP exposure in the saline solution of the studied calculated concentration and saline solutions S-22 and S-24.
2) The second series: we assessed the effect of the pre-incubation of neurons in 4-AP saline solutions with the concentration of 1·10^−12^ M and S-24 on the EC of neurons after exposure to 4-AP in the concentration of 1·10^−2^ M that is commonly used in electrophysiology.

For each neuron, EC was recorded three times: 1) before the exposure (neuron placed in saline solution), 2) after 30 min incubation in the 4-AP systems with the concentration of 1·10^−12^ M or S-24, 3) after 20 min of exposure to 4-AP at the standard concentration of 1·10^−2^ M.

The measurements were done at room temperature (18–21°C) using intracellular glass microelectrodes filled with 2.5 M of KCl and with 10–30 MΩ resistance. The recorded parameters were: membrane resting potential (*Vm*), i.e., the initial *Vm* value before the start of a series of tactile stimuli or electrical stimulation and its value during the experiment; the action potential amplitude (*Vs*), the generation threshold (threshold potential) of the evoked action potentials (*Vt*), and duration of the action potentials (*ts*) of premotor interneurons of defensive behavior. From the amplifier output, the signal was recorded in a digital form on the computer, with its subsequent processing. Quantization frequency during recording was 200 μs. Since the premotor interneurons LPa3, RPa3, LPa2, and RPa2 are normally silent, in order to call the action potential in an isolated substance, a 1 s rectangular-shaped current pulse was applied through a recording microelectrode to the cell; the selected stimulation current magnitude was minimal for generation of the action potential. In this case, the minimum current required for generation of two to three action potentials was used. Typically, this current strength is within the range of 1.7–3.5 nA. In each study group, 8–10 neurons were analyzed. The volume of the bath into which a ganglia nerve ring was placed was 2 ml. Replacement of solutions was carried out by a gradual substitution of a previous solution by a new one (Andrianov et al., 2015).

Statistical analysis was performed using Microsoft Excel software. The significance of differences was assessed by the Student's t-test. The ECs of each neuron kept in the studied 4-AP systems were normalized to the EC in saline according to the Equation 1.

$$\mathrm{EC}\;\%\;=\;\frac{EC_{i}}{\;{\mathrm{EC}}_{\mathrm{SS}}}\times 100\%,$$ (1)

where:

EC_*i*_–the EC value after the exposure of the neuron to the 4-AP system of a certain concentration, EC_SS_–the value of the EC of the control neuron, i.e., incubated in saline solution without keeping it in the 4-AP systems.

## Results and Discussion

**FIGURE 1:**
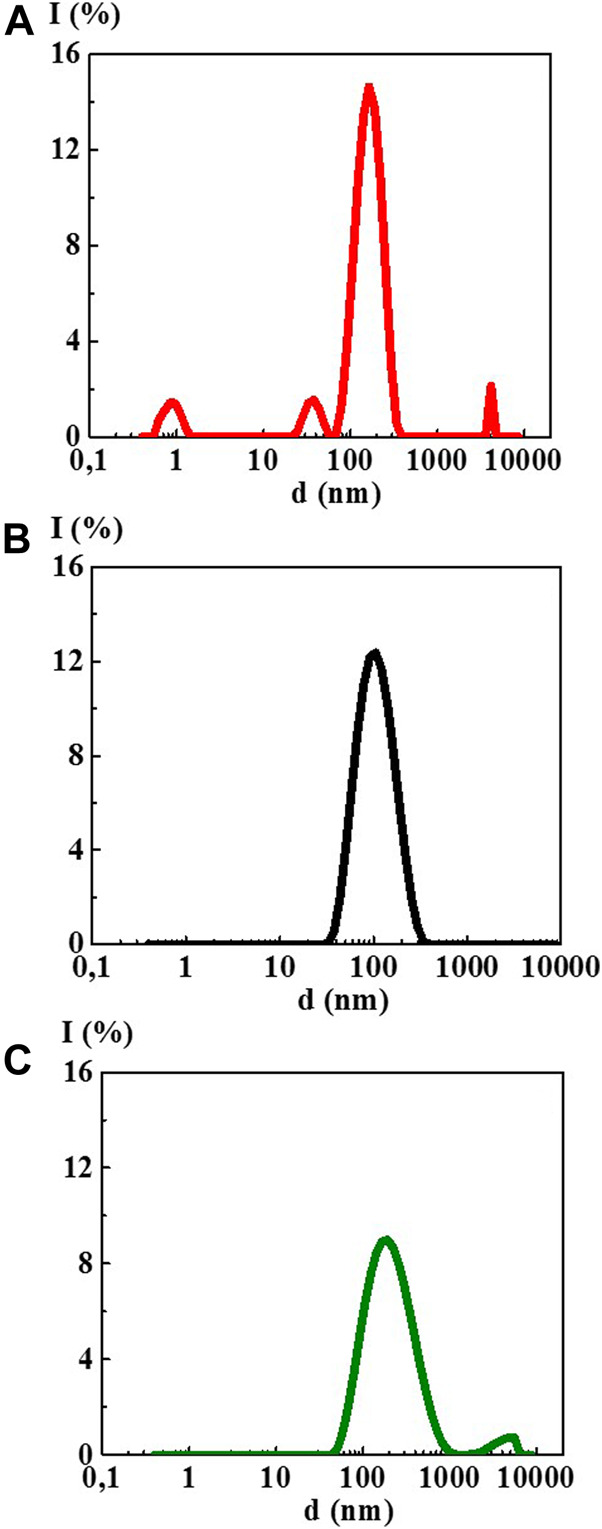
Particle size distribution based on light scattering intensity of the 4-AP systems: **(A)** 1·10^−2^ М, **(B)** 1·10^–20^ М, **(C)** S-24. Measurements were performed at 25 ± 0.1°C.

The study of 4-AP aqueous solutions within a wide range of the calculated concentrations 1·10^−20^–1·10^−2^ M by the DLS method showed that as dilution proceeds, the solutions self-organized with the formation of complex dispersed systems. At the concentrations of 1·10^–2^ (Figure 1A) and 1·10^−4^ M, there was still no sufficiently formed dispersed phase of a certain size in 4-AP solutions as was evidenced by a high value of the polydispersity index (IP) equal to 0.65 and 0.55, respectively (Supplementary Table S1). At 1·10^−4^ M, formation of a dispersed phase-like domain, hundreds of nm in size, probably just starts.

Starting from 1·10^−6^ M and up to 1·10^−20^ M (Figure 1B, Supplementary Figure S1), 4-AP solutions are complex dispersed systems. The monomodal size distribution of the light scattering intensity was observed almost in all concentration intervals, which indicates the predominant formation of domains or nanoassociates hundreds of nanometers in size (see Figure 1B). The IP of the 4-AP systems ranged from 0.29 to 0.45 (Supplementary Table S1). Similar to other research (Konovalov and Ryzhkina, 2014; Ryzhkina et al., 2018a; Ryzhkina et al., 2018b; Ryzhkina et al., 2019; Ryzhkina et al., 2020), this concentration range of the 4-AP systems can be divided into two intervals in which domains (1·10^−6^–1·10^−10^ M) and nanoassociates (1·10^−12^–1·10^−20^ M) form. Concentrations of 1·10^−10^ and 1·10^−12^ M are the threshold levels. In the vicinity of such concentrations, significant changes in the properties of self-organized systems are often observed (Konovalov and Ryzhkina, 2014; Ryzhkina et al., 2018a; Ryzhkina et al., 2018b; Ryzhkina et al., 2019; Ryzhkina et al., 2020).

The study of aqueous solutions S-22 and S-24 by the DLS method showed a bimodal particle size distribution in both cases (Figure 1C) with IP > 0.5 (Supplementary Table S1) which does not make it possible to correctly determine the particle size ranging from tens to thousands of nm and to classify these solutions as dispersed systems with full confidence.

**FIGURE 2:**
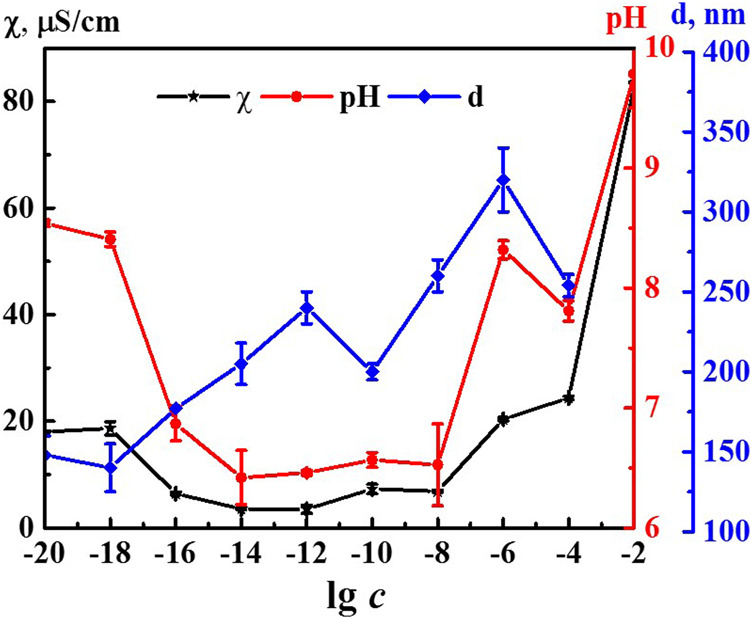
Dependence of particle size (d), specific conductivity (χ), and рН on concentration (c, M) of the 4-AP systems. Measurements were performed at 25 ± 0.1°C.

It is known that formation and rearrangement of the dispersed phase initiates emergence of nonmonotonic concentration dependences of physicochemical properties of the systems (Konovalov and Ryzhkina, 2014; Ryzhkina et al., 2018a; Ryzhkina et al., 2018b; Ryzhkina et al., 2019; Ryzhkina et al., 2020). Figure 2 shows concentration dependences of the size of nanoassociates (*d*), specific electrical conductivity χ, and pH of the 4-AP systems. The size of domains at 1·10^−4^ M (IP > 0.5) should be considered as estimated. The χ and pH dependences are practically correlated with maximum at concentrations of 1·10^−20^, 1·10^−18^, and 1·10^−6^ M and a minimum within the range of 1·10^−14^–1·10^−8^ M (to learn more about the underlying cause of the observed correlation, please refer to the Supplementary Material). Dependence *d* is of a similar nonlinear nature. It also has a plateau within the range of 1·10^−20^–1·10^−18^ M and the maximum at 1·10^−6^ M. In contrast to the χ and pH dependences, the *d* curve contains an additional maximum at 1·10^−12^ M below which dependences of physicochemical properties and *d* are highly anticorrelated. Thus, rearrangement of domains and nanoassociates in the self-organized 4-AP systems is accompanied by a consistent change in physicochemical properties - electrical conductivity and pH with extremes at critical concentrations of 1·10^−18^, 1·10^−12^, and 1·10^−6^ M.

**FIGURE 3:**
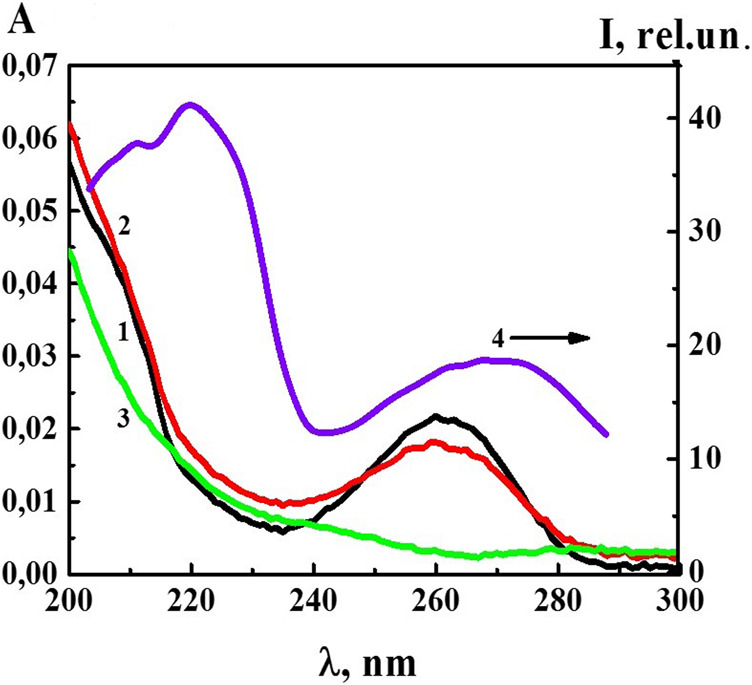
The absorption spectra of the 4-AP systems: 1) 1·10^−6^ М, 2) 1·10^−12^ М, 3) S-24, and the excitation spectra (*λ*_em_340 nm) of the 4-AP system at 4) 1·10^−6^ М. Measurements were performed at 25 ± 0.1°C.

At the next stage of work, we studied the UV absorption and fluorescence spectra of the 4-AP systems. UV absorption spectra (A) (Figure 3) of the 4-AP systems in the range of calculated concentrations from 1·10^−6^ M to 1·10^−20^ M (water used as a solvent for the preparation of solutions was used as a reference sample) had a maximum at 260 nm and a shoulder in the 210–220 nm region, similar to the previously described BAS systems in similar ranges of calculated concentrations (Ryzhkina et al., 2018a; Ryzhkina et al., 2018b). The data obtained are consistent with the results (Ryzhkina et al., 2018a; Ryzhkina et al., 2018b) which found that appearance of optical absorption in the region of the indicated wave lengths, while the systems are diluted, was associated with formation of the dispersed phase (domains and nanoassociates). As for the S-22 and S-24 systems, their absorption curve (curve 3) was practically indistinguishable from the curve of water used for dilution in the 230–280 nm range, although in the high frequencies range around 210 nm the absorption was higher than that of water. The DLS method showed that at such high dilutions the systems were less ordered than within the range from 1·10^−6^ M to 1·10^−20^ M (Figure 1).

**FIGURE 4:**
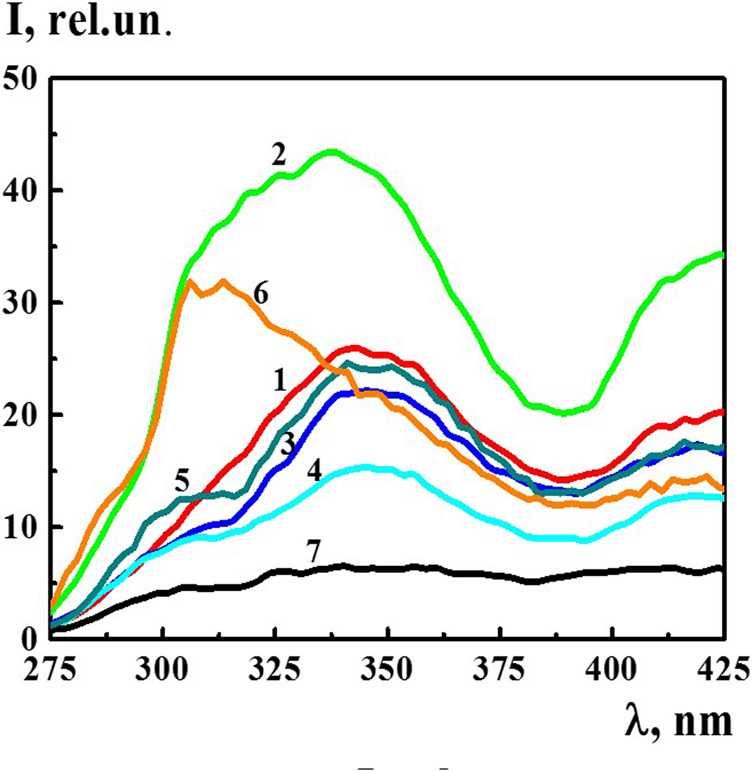
The fluorescence spectra (*λ*_ex_225 nm) of the 4-AP systems at 1) 1·10^−8^ М, 2) 1·10^−12^ М, 3) 1·10^−16^ М, 4) 1·10^−20^ М, 5) S-22, 6) S-24, and 7) diluted double distilled water (fourth centesimal dilution corresponding to 1·10^−8^ M of the 4-AP system). Measurements were performed at 25 ± 0.1°C.

The fluorescence spectra of the 4-AP systems (*λ*_ex_ 225 nm) within the concentration range of 1·10^−20^–1·10^−6^ M, in which domains and nanoassociates formed, had broad overlapping bands in two spectral ranges of 300–375 and 400–450 nm (see for example Figure 4). In addition, in a wide range of wavelengths, the spectra had second order Rayleigh scattering peaks at 450 nm and Raman scattering peaks at 490 nm (see Supplementary Figure S2). The presence of such peaks in the fluorescence spectra is known to be typical for systems with very low fluorophore concentrations and for systems containing highly scattering nanoparticles (Jameson, 2014). Indeed, the DLS method has shown that nanoassociates of hundreds of nanometers in size form in the 4-AP systems in the studied concentration interval (Figure 1).

In the spectrum of bidistilled water used for solution preparation, the bands within the range of 275–425 nm (*λ*_ex_ 225) were absent. Figure 4 shows the fluorescence spectra (*λ*_ex_ 225 nm) of the 4-AP systems with the concentration of 1) 1·10^−8^, 2) 1·10^−12^, 3) 1·10^−16^, 4) 1·10^−20^ M, systems 5) S-22, 6) S-24, and 7) bidistilled water in the fourth centesimal dilution corresponding to 1·10^−8^ M of the 4-AP system. It is apparent that a short-wavelength band with the maximum at 340 nm was dominant and more distinctive. As the systems were being diluted, starting from 1·10^−16^ M and further, a slightly pronounced shoulder appeared in the region of 310 nm on the 340 nm band which was clearly visible in the S-22 system (curve 5) while maintaining an overall shape of the band. The fluorescence spectrum of the S-24 system had a different band shape with the maximum in the region of 312–325 nm. This suggests that the nature of the structures formed at high dilutions differs from the nature of domains and nanoassociates formed, as a rule, in the 1·10^−4^–1·10^−18^ M range (Sedlák, 2006; Sedlák and Rak, 2013; Elia et al., 2014; Ryzhkina et al., 2018a; Ryzhkina et al., 2018b; Patsaeva et al., 2018; Ryzhkina et al., 2019; Ryzhkina et al., 2020), i.e., capable of containing a certain amount of solute (Ryzhkina et al., 2017).

The nonmonotonic character of changes in the fluorescence spectra of the 4-AP systems within the range of 1·10^−20^–1·10^−6^ M suggests that the 340 nm band (*λ*_ex_ 225 nm) may be associated with formation and rearrangement of the domains and nanoassociates formed in diluted 4-AP systems.

Figure 3 shows the excitation spectrum (*λ*_em_ 340 nm) of the 4-AP system with the concentration of 1·10^−6^ M (spectrum 4). The spectrum consisted of two weakly overlapping broad bands in the short-wavelength (212–240 nm) and long-wavelength regions (250–290 nm) with the maximum at 212–230 and 260–280 nm. It was clear that the short-wavelength band with a maximum at 220 nm was 2.5 times more intense than the long-wavelength band with the maximum at 270 nm. Excitation and absorption spectra were close, and this is typical for fluorescent systems (Jameson, 2014).

The fluorescence band at 340 nm (at *λ*_ex_ 225 nm) was dominant in a wide concentration range in which nanoassociates were formed, which determined the properties of the dilute systems (Konovalov and Ryzhkina, 2014; Ryzhkina et al., 2018a; Ryzhkina et al., 2018b; Ryzhkina et al., 2019; Ryzhkina et al., 2020). Therefore, it will be most informative to analyze the intensity of this particular band in order to establish the relationship between self-organization in the 4-AP systems and fluorescence intensity.

**FIGURE 5:**
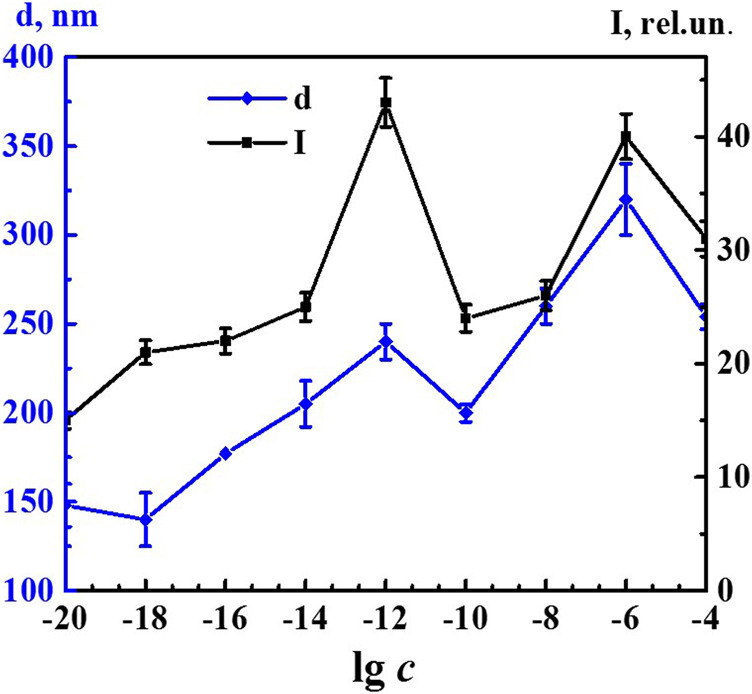
Dependence of particle size (d) and fluorescence intensity (*λ*_ex_225 nm, *λ*_em_340 nm) (I) on concentrations (*c*/M) of 4-AP. Measurements were performed at 25 ± 0.1°C. Data presented as the mean value ±standard deviation.

Figure 5 shows nonmonotonic concentration dependency of the fluorescence intensity of the 340 band (*λ*_ex_ 225 nm) and concentration dependency of the size of nanoassociates. Both dependences had a similar trend with well-pronounced extremes at critical concentrations of 1·10^−12^ and 1·10^−6^ M and a small plateau within the range of 1·10^−20^–1·10^−18^ M. The concentration dependency trend presented in this figure confirms the suggestion made in other research (Konovalov and Ryzhkina, 2014; Ryzhkina et al., 2018a; Ryzhkina et al., 2018b; Ryzhkina et al., 2019; Ryzhkina et al., 2020) about the decisive role of the formation and rearrangement of nanoassociates in the coherent nonmonotonic change in the properties of self-organized dilute dispersed systems. In this work, using 4-AP as an example, we established that these properties include not only the size of nanoassociates (*d*), specific electrical conductivity (χ), and pH of solutions (Figure 2), but also fluorescence intensity (Figure 5).

One of the goals of this work was to research the self-organization and properties of aqueous mixed systems (MS) prepared from solutions with high and low calculated concentrations of 4-AP or using highly diluted systems such as S-24. For this purpose, we studied mixed systems containing a solution of 4-AP at a constant concentration of 1·10^–2^ M as the first component, since this is the concentration that is usually used for electrophysiological studies. As the second component, we chose the dispersed systems in which 4-AP concentration varied (1·10^−6^, 1·10^−12^, and 1·10^−18^ M) or the S-24 system.

**FIGURE 6:**
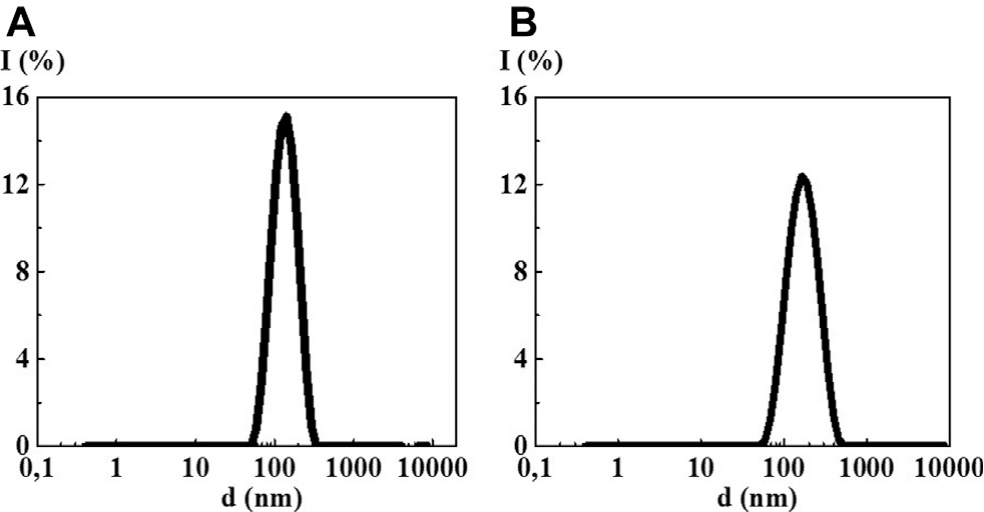
Particle size distribution based on light scattering intensity in the 4-AP mixed systems: **(A)** 10^−2^/10^−12^ М, **(B)** 10^−2^ М/S-24. Measurements were performed at 25 ± 0.1°C.

The study of MS 10^−2^/10^−6^, MS 10^−2^/10^−12^, MS 10^−2^/10^−18^, and MS 10^−2^/S-24 by the DLS method in all cases showed formation of new dispersed systems with a monomodal particle size distribution (Figure 6, Supplementary Figure S3) with the IP less than that of the original systems (from 0.25 to 0.40) indicating their higher ordering (See Supplementary Table S2). However, it is not possible to confirm formation of a new dispersed system with new properties by the methods of UV absorption and fluorescence spectroscopy because the first component concentration is too high for these sensitive spectral methods (Supplementary Figures S4, S5).

Within the framework of the hypothesis proposed in our previous works (Konovalov and Ryzhkina, 2014; Ryzhkina et al., 2018a; Ryzhkina et al., 2018b; Ryzhkina et al., 2019; Ryzhkina et al., 2020), it is suggested that the nonmonotonous concentration dependencies of the parameters of nanoassociates and physicochemical properties of the systems in the low concentrations range can be used to predict the emergence of the nonmonotonic concentration dependencies of the bioeffects.

In this case, we may assume that the 4-AP-based individual and mixed systems are able to nonmonotonically affect biological objects within the range of the concentrations at which domains and nanoassociates form.

To confirm this assumption, we chose terrestrial snail (*Helix lucorum*) neurons as a biological object. However, they were able to function only in a saline solution. Works (Ryzhkina et al., 2012; Murtazina et al., 2014) show that regularities of physicochemical behavior of aqueous systems of BAS with low concentrations persist in salt systems. We have established experimentally that the results of studying the self-organization and physicochemical properties of aqueous systems of BAS with low concentrations can be used to predict the bioeffects of salt systems of the same BAS within the same concentration range (Ryzhkina et al., 2012; Murtazina et al., 2014). In this regard, we can assume that the systems selected for research - 4-AP in the range of low concentrations (1·10^−6^–1·10^−20^ M) and systems S-22 and S-24 - will create a nonmonotonic effect on the electrical characteristics of the terrestrial snail neurons in the salt systems.

**FIGURE 7:**
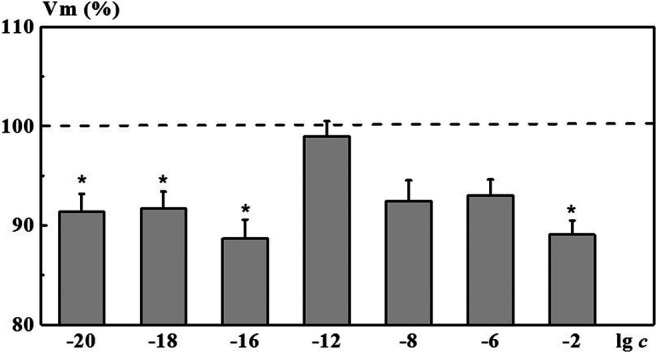
Change in the membrane resting potential (*Vm*) of command neurons upon action of the 4-AP systems. The *x*-axis represents the logarithm of 4-AP concentration. The *y*-axis represents *Vm* value after exposure to the corresponding 4-AP system normalized to *Vm* value in saline, %. Dotted line denotes *Vm* level in control taken as 100%. Data presented as the mean value ±standard error of the mean. *Statistically significant difference vs control neurons (i.e., without exposure to the 4-AP systems) (*p* < 0.05).

Indeed, after incubation of neurons in the 4-AP salt systems, we found a nonmonotonic character of concentration dependence of the membrane resting potential *Vm*. As shown in Figure 7, a solution of 4-AP with the concentration of 1·10^−2^ M, standard for electrophysiology, reduced the value of the membrane resting potential *Vm* (depolarization shift) by 10.9% relative to the membrane potential in saline solution (*ΔVm* = 10.9%). Under the influence of the 4-AP systems with low calculated concentrations of 1·10^−16^, 1·10^−18^, and 1·10^−20^ M, a statistically significant change in the membrane potential of neurons relative to the membrane potential in saline solution was observed, amounting to 8.3–11.3%. This change is comparable or even more pronounced than the one found when neurons were exposed to 1·10^−2^ M of 4-AP solution. The influence of the 4-AP saline systems with the concentrations of 1·10^−6^ and 1·10^−8^ M as well as the influence of saline solutions S-22 and S-24 on *Vm* was less pronounced (data for S-22 and S-24 are presented in of the Supplementary Table S1). These systems reduced the *Vm* value by 7.0, 7.5, 7.6, and 6.5%, respectively. In this case, the studied systems did not cause changes in the magnitude of the evoked action potentials *Vt*. The 4-AP system with the concentration of 1·10^−12^ M practically did not affect electrical characteristics of identified neurons.

**FIGURE 8:**
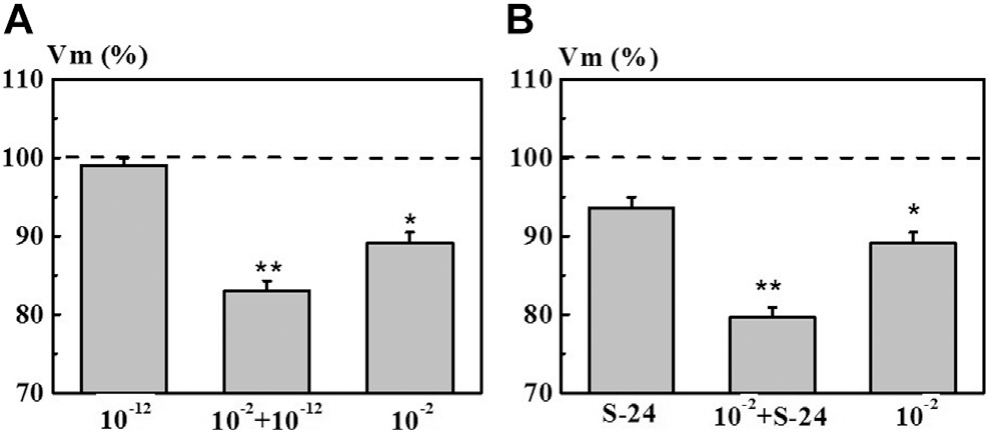
Value of membrane resting potential (*Vm*) of command neurons upon action of the mixed 4-AP systems. Panel **(A)**: systems at 1·10^−2^ M, 1·10^−12^ M, and their mixed system “10^−2^ M + 10^−12^ M”. Panel **(B)**: systems at 1·10^−2^ M, S-24, and their mixed system “10^−2^ M + S-24”. Various types of solutions are listed along the *x*-axis. The *y*-axis represents *Vm* value after exposure to the corresponding 4-AP system normalized to *Vm* value in saline, %. Dotted line denotes the initial level of *Vm* taken as 100%. Data presented as the mean value ±standard error of the mean. Statistically significant difference vs control neurons (i.e., without exposure to the 4-AP systems) (*p < 0.05 and **p < 0.01).

The second series of experiments was aimed at elucidating the effect of pre-incubation of neurons in the 4-AP system with the concentration of 1·10^−12^ M and in the S-24 system on the EC of the neurons exposed to 4-AP with the concentration of 1·10^−2^ M which is common for electrophysiology (Figure 8, columns 10^−2^ + 1·10^−12^ M and 10^−2^ + S-24). It was found that pre-incubation of neurons in the 4-AP system with the concentration of 1·10^−12^ M and in the S-24 system lead to a synergistic decrease in the membrane potential *Vm* by 17.0 and 21.0%, respectively, due to a subsequent exposure to 4-AP solution with the concentration of 1·10^−2^ M (Figure 8). However, neither the action of individual 4-AP systems nor the action of 4-AP (1·10^−2^ M) after pre-incubation in 1·10^−12^ M or in the S-24 system did cause changes in the *Vt* value.

Thus, it was found that self-organized dilute dispersed 4-AP systems have a significant modifying effect on the electrical characteristics of neurons. An additional confirmation of that is the revealed synergistic decrease in the membrane potential when the neuron is subsequently exposed to a 4-AP solution at a concentration of 1·10^−2^ M. It is possible that the 4-AP systems with a low calculated concentration cause a change in the conformation and/or charge in the active center of the K+ channel, which alters the efficacy of binding of the blocker to the channel. A similar result was obtained in other works (Epstein et al., 2003; Marotta et al., 2003) showing that pretreatment of the cerebellum and sections of the rat hippocampus as well as the cochlear ganglia with BAS solutions with a low calculated concentration results in modifying the effects of high concentration of these BAS on biological objects.

Comparing the nonmonotonic concentration dependences of the membrane resting potential *Vm* (Figure 7) with the dependences of the size of the dispersed phase *d* and the physicochemical properties of the system (Figure 2), we can note their interrelation. The extreme values of *d*, pH, χ, and changes in the bioeffect were found for the same critical concentrations. However, the behavior of these dependences was different: in the vicinity of 1·10^−6^ and 1·10^−18^ M the *Vm*, pH, and *χ* have high values, and at 1·10^−12^ M - they are minimal, while the values of *d* are maximum at 1·10^–6^ and 1·10^−12^ and minimum at 1·10^−18^ M. The data obtained make it possible to suggest that change in the pH of the medium caused by rearrangement of the dispersed phase can play a significant role in the bioeffect occurrence.

In other research (Tikhonov et al., 2019; Serova et al., 2020), it is noted that a change in the pH of extracellular environment in the nervous system causes various cellular responses with participation of ion channels; it was also shown that even small changes in pH affect excitability of neurons. The authors of these works concluded that local shifts in the environmental acidity are one of the key factors regulating neuronal activity. One work (Konovalov et al., 2017) establishes the relationship between the rearrangement of nanoassociates, which causes significant shifts in the pH of the medium, and the nonmonotonic dependences of structural changes in the synaptosome membranes and endoplasmic reticulum of mouse brain cells.

An analysis of the obtained results and published data allows us to conclude that a consistent change in the nature and parameters of the dispersed phase, as well as the pH of the medium, apparently determine the nonmonotonic nature of the effect of the 4-AP systems in a 1·10^−20^–1·10^−6^ M concentration range on the resting membrane potential of neurons.

As for mixed systems (MS) prepared from solutions with a high concentration of 4-AP and dilute dispersed 4-AP systems, such systems combine the advantages associated with a quite high concentration of the active ingredient (4-AP) and the structuredness of dispersed diluted systems. Dilute dispersed BAS systems are known to be capable of modifying the physicochemical properties of bio-membranes (Konovalov and Ryzhkina, 2014; Konovalov et al., 2017). Such systems are an effective tool for regulating the effect of biologically active substances on biological objects; they can reduce doses, toxicity, and expand the spectrum of action of biologically active substances.

## Conclusion

Thus, it was demonstrated that aqueous solutions of the potassium channel blocker 4-aminopyridine (4-AP) within the range of calculated concentrations of 1·10^−20^–1·10^–6^ M are self-organized dispersed systems. The relationship was established between the nonmonotonic concentration dependences of the dispersed phase size, specific electrical conductivity, the pH, and fluorescence intensity (*λ*_ex_ 225 nm, *λ*_em_ 340 nm) of the aqueous 4-AP systems. The coherence of these dependences indicates that the fluorescent and physicochemical properties are caused by the rearrangement of domains and nanoassociates. For the first time, the nonmonotonic effect of 4-AP saline solutions within the range of low concentrations on electrical properties of identified neurons of the *Helix lucorum* snail was predicted and experimentally verified. It was demonstrated that the 4-AP systems with concentrations of 1·10^−20^–1·10^−6^ M cause a statistically significant nonmonotonic decrease in the membrane resting potential of neurons amounting to 7.0–11.3% relative to the membrane potential in saline.

An analysis of the obtained results and published data allows us to conclude that a consistent change in the nature and parameters of the dispersed phase, as well as the pH of the medium, apparently determine the nonmonotonic nature of the effect of the 4-AP systems in a low concentration range on the resting membrane potential of neurons. It was found that as a result of preliminary incubation of neurons in the 4-AP system with a concentration of 1·10^−12^ M and the S-24 system, subsequent exposure to a 4-AP solution at a concentration of 1·10^−2^ M caused a synergistic decrease in the membrane potential by 17.0% and 21.0%, which indicates a significant modifying effect exerted on neurons of dilute dispersed 4-AP systems. The revealed ability to form nanoassociates in the range of low calculated concentrations may be the key to understanding the mechanism by which the 4-AP systems affect biological objects and can be utilized for developing approaches to more efficient and safe clinical use of 4-AP.

## Data Availability

The original contributions presented in the study are included in the article/Supplementary Material, further inquiries can be directed to the corresponding author.

## Footnotes

1 For the reasoning behind the usage of the “calculated concentration” term please refer to parts *Materials and Methods*, *Experimental Design*).
