# Supplementary material for: Diluted Aqueous Dispersed Systems of 4-Aminopyridine: The Relationship of Self-Organization, Physicochemical Properties, and Influence on the Electrical Characteristics of Neurons
Source: Front Chem. 2021 Mar 16;9:623860. doi: 10.3389/fchem.2021.623860 (PMC8007878; doi:10.3389/fchem.2021.623860)
Supplement: Supplementary file 1 [file Table1.DOCX]

Supplementary Material

|  |
| --- |
|  |
|  |

# Supplementary Figure 1. Correlation function (a) and particle size distribution based on light scattering intensity (b) and volume (c) of the 4-AP system at a concentration of 1·10^-20^ М. Measurements were performed at 25±0.1 °C.

**TABLE 1ǀ** The polydispersity index (IP) of the 4-AP systems.

| Conc, M | 1·10^-2^ | 1·10^-4^ | 1·10^-6^ | 1·10^-8^ | 1·10^-10^ | 1·10^-12^ | 1·10^-14^ | 1·10^-16^ | 1·10^-18^ | 1·10^-20^ | S-24 |
| --- | --- | --- | --- | --- | --- | --- | --- | --- | --- | --- | --- |
| IP | 0.65 | 0.55 | 0.4 | 0.45 | 0.36 | 0.45 | 0.29 | 0.29 | 0.38 | 0.45 | 0.54 |

In a series of our previous works /1-4/, we have used a special container (μ-metal) that protects the contents from low-frequency electromagnetic fields to prove that the emergence of consistent non-monotonic concentration dependences of specific conductivity and pH of diluted dispersed systems is associated with the formation and rearrangement of nanoassociates, which in turn depends on the structure of the dissolved substance and the presence of external low-frequency electromagnetic fields. In the absence of low-frequency electromagnetic fields nanoassociates are not formed and unique non-monotonic interrelated properties of systems do not emerge, i.e. no low-frequency electromagnetic fields - no nanoassociates - no non-monotonic changes in properties.

Thus, speaking of the relationship between specific conductivity and pH (other physicochemical properties were not studied in the presented manuscript), it should be kept in mind that the main reason for the interconnected relationship of the physicochemical properties of self-organized diluted dispersed systems lies in their coherence with the parameters of nanoassociates.

Works /1-4/ show that most often the most significant correlation (r˃0.8, p<0.05) in the range of calculated concentrations 10^-8^ - 10^-18^ M is observed between the ζ-potential of nanoassociates and the specific electrical conductivity of systems. It is known that the ζ-potential is part of the electrical double layer (EDL), i.e., a self-organized ordered structure at the phase boundary in which the potential-determining ions and counterions are arranged in a certain order /5/. Since nanoassociates are formed mainly by water structures and the ζ-potential of nanoassociates is always negative /1-4/, hydroxy ions are most likely the potential-determining ions and the counterions are hydroxonium ions. This means that the reorganization of nanoassociates as they dilute can be accompanied by a change in the content of hydroxonium and hydroxy ions both in the electrical double layer and in bulk water, i.e., by a change in the pH of the medium.

Thus, the change in the specific conductivity of diluted dispersed systems is the result of complex processes associated with the formation of charged nanoassociates and changes in the content of hydroxy and hydroxonium ions in the medium, which may explain the observed relationship between the nonmonotonic concentration dependences of specific conductivity and pH.

Since the question of justification of the structure of nanoassociates is currently only starting to be discussed /6/, it is probably premature to explain in more detail the observed relationship between the nonmonotonic concentration dependences of specific conductivity and pH. Therefore, in this paper, similarly to /7/, we limited ourselves to stating the observed relationship between the nonmonotonic concentration dependences of specific conductivity and pH. Similarly, to the authors of this paper, we also believe that the correlations between widely varying variables indicate that a single cause underlies the phenomena, i.e., in our case, the cause is the rearrangement of nanoassociates with dilution.

The study of 4-AP systems by the electrophoretic light scattering method using a Zetasizer Nano ZSP analyzer (Malvern Instruments, UK) showed that the ζ-potential distribution did not meet the quality criterion, so it is not given in the article. However, this does not mean that nanoassociates are devoid of EDL, it could mean that the EDL in the 4-AP systems is "fuzzy," "loose," not well ordered. As repeatedly noted /1,4/, the structure of the dissolved substance has a significant impact on the quality of the size distribution and zeta potential.

1. Ryzhkina IS, Kiseleva YuV, Murtazina LI, Mishina OA, Sherman ED, Konovalov AI. Comparative study of self-organization and physicochemical properties of highly diluted aqueous solutions of phenol bioantioxidants. *Doklady Physical Chemistry* (2012) **447**: 203–206. <https://doi.org/10.1134/S0012501612110036>
2. Ryzhkina IS, Kiseleva YuV, Mishina OA, Timosheva AP, Sergeeva SYu, Kravchenko AN, Konovalov AI. Correlations between the self-organization, physicochemical properties and biological activity of Mebicar in dilute aqueous solutions. *Mendeleev Commun*. (2013) **23**: 262–264. <https://doi.org/10.1016/j.mencom.2013.09.008>
3. Konovalov A, Ryzhkina I, Maltzeva E, Murtazina L, Kiseleva Yu, Kasparov V. Nanoassociate formation in highly diluted water solutions of potassium phenosan with and without permalloy shielding. *Electromagn. Biol. Med*. (2015) **34** (2): 141–146. <https://doi.org/10.3109/15368378.2015.1036070>
4. Konovalov AI, Ryzhkina IS. Formation of nanoassociates as a key to understanding of physicochemical and biological properties of highly dilute aqueous solutions. Russ. Chem. Bull. (Int. Ed.) (2014) 63: 1-14. doi.org/10.1007/s11172-014-0388-y
5. Hunter RJ. (1981). Zeta-Potential in Colloid Science: Principles and Applications. <https://doi.org/https://doi.org/10.1016/C2013-0-07389-6>

6. Yinnon TA. Liquids Prepared by Serially Diluting and Vigorously Shaking of Aqueous Solutions: Unveiling Effects of the Solute on their Properties. WATER (2020) **10:**115. <https://waterjournal.org/uploads/vol10/yinnon-2/WATER.2019.2.Yinnon.pdf>

7. Elia V, Oliva R, Napoli E, Germano R, Pinto G, Lista L, Niccoli M, Toso D, Vitiello G, Trifuoggi M, Giarra A, Yinnon TA. Experimental study of physicochemical changes in water by iterative contact with hydrophilic polymers: A comparison between Cellulose and Nafion. *J. Mol. Liq.* (2018) 268: 598-609. <https://doi.org/10.1016/j.molliq.2018.07.045/>

**TABLE 2ǀ**T he polydispersity index (IP) of the 4-AP mixed systems.

| Conc, M | 10^-2^/10^-6^ | 10^-2^/10^-12^ | 10^-2^/10^-18^ | 10^-2^/S-24 |
| --- | --- | --- | --- | --- |
| IP | 0.33 | 0.29 | 0.33 | 0.38 |

**Supplementary Figure 2.** The fluorescence spectra (λ_ex_225 nm) of the 4-AP systems at 1·10^-8^ М. Measurements were performed at 25±0.1 °C.

|  |  |
| --- | --- |

**Supplementary Figure 3.** Particle size distribution based on light scattering intensity in the 4-AP systems: (a) 1·10^-2^ М (red line), 1·10^−12^ М (blue line), 10^-2^ М/10^-12^ М (black line), (b) 1·10^-2^ М (red line), S-24 (blue line), 10^-2^ М/S-24 (black line). Measurements were performed at 25±0.1 °C.

**Supplementary Figure 4.** The fluorescence spectra (λ_ex_225 нм) of the 4-AP systems at a concentration of (1) 1·10^-2^ М, (2) 1·10^-12^ М and (3) mixed system10^-2^М/10^-12^М. Measurements were performed at 25±0.1 °C.

|  |  |
| --- | --- |

**Supplementary Figure 5.** (a) UV and (b) fluorescence spectra (λ_ex_225 nm) of the 4-AP systems at a concentration of (1) 1·10^-2^М, and (2) mixed system 10^-2^ М/10^-12^ М. Measurements were performed at 25±0.1 °C.

**Table 3|** Value of membrane resting potential (*Vm*) of command neurons upon action of the 4-AP systems and its changes regarding control. The initial level of *Vm* in saline solution– 100%.

| Concentration of 4-AP, M | ***Vm*** | |
| --- | --- | --- |
|  | ***Vm*, %** | **Δ*Vm*, %** |
| **1·10^-2^** | 89,1±1.4 | 10.9 |
| **1·10^-6^** | 93.0±1.6 | 7.0 |
| **1·10^-8^** | 92.4±2.08 | 7.6 |
| **1·10^-12^** | 99.5±1.5 | 0.5 |
| **1·10^-16^** | 88.7±1.9 | 11.3 |
| **1·10^-18^** | 91.7±1.67 | 8.3 |
| **1·10^-20^** | 91.3±1.86 | 8.7 |
| **S-22** | 92.3±2.01 | 7.7 |
| **S-24** | 93,6±1,5 | 6.4 |
| **1·10^-2^ + 1·10^-12^** | 83.0±1,3 | 17 |
| **1·10^-2^ + S-24** | 79.0±1,5 | 21 |
